# Transcriptome database resource and gene expression atlas for the rose

**DOI:** 10.1186/1471-2164-13-638

**Published:** 2012-11-20

**Authors:** Annick Dubois, Sebastien Carrere, Olivier Raymond, Benjamin Pouvreau, Ludovic Cottret, Aymeric Roccia, Jean-Paul Onesto, Soulaiman Sakr, Rossitza Atanassova, Sylvie Baudino, Fabrice Foucher, Manuel Le Bris, Jérôme Gouzy, Mohammed Bendahmane

**Affiliations:** 1 Reproduction et Développement des Plantes UMR INRA-CNRS- Université Lyon 1-ENSL, Ecole Normale Supérieure, 46 allée d'Italie, Lyon Cedex 07, 69364, France; 2 INRA, Laboratoire des Interactions Plantes-Microorganismes (LIPM), UMR441, Castanet-Tolosan, F-31326, France; 3 CNRS, Laboratoire des Interactions Plantes-Microorganismes (LIPM), UMR2594, Castanet-Tolosan, F-31326, France; 4 Laboratoire BVpam, EA2061, Université de Saint-Etienne, Université de Lyon, rue du Dr Michelon, Saint-Etienne, F-42023, France; 5 INRA, Unité de Recherches Intégrées en Horticulture, 400 route des Chappes BP 167, Sophia Antipolis Cedex, 06903, France; 6 Institut de Recherche en Horticulture et Semences (INRA, Agrocacmpus-Ouest, Université d’Angers), SFR 149 QUASAV, Beaucouzé cedex, BP 60057-49071, France; 7 Université de Poitiers, UMR CNRS 7267 Écologie et Biologie des Interactions, 40 Av. du Recteur Pineau, Poitiers Cedex, 86022, France; 8 Institut Méditerranéen de Biodiversité et d’Ecologie marine et continentale, UMR Université d'Aix-Marseille- CNRS 7263, Université d’Aix-Marseille, IRD 237, Université d’Avignon, Avenue Escadrille Normandie-Niemen, Marseille, F-13397, France

**Keywords:** Rose, Transcriptome, Gene expression atlas

## Abstract

**Background:**

For centuries roses have been selected based on a number of traits. Little information exists on the genetic and molecular basis that contributes to these traits, mainly because information on expressed genes for this economically important ornamental plant is scarce.

**Results:**

Here, we used a combination of Illumina and 454 sequencing technologies to generate information on *Rosa sp.* transcripts using RNA from various tissues and in response to biotic and abiotic stresses. A total of 80714 transcript clusters were identified and 76611 peptides have been predicted among which 20997 have been clustered into 13900 protein families. BLASTp hits in closely related Rosaceae species revealed that about half of the predicted peptides in the strawberry and peach genomes have orthologs in *Rosa* dataset. Digital expression was obtained using RNA samples from organs at different development stages and under different stress conditions. qPCR validated the digital expression data for a selection of 23 genes with high or low expression levels. Comparative gene expression analyses between the different tissues and organs allowed the identification of clusters that are highly enriched in given tissues or under particular conditions, demonstrating the usefulness of the digital gene expression analysis. A web interface *ROSAseq* was created that allows data interrogation by BLAST, subsequent analysis of DNA clusters and access to thorough transcript annotation including best BLAST matches on *Fragaria vesca, Prunus persica* and *Arabidopsis*. The rose peptides dataset was used to create the *ROSAcyc* resource pathway database that allows access to the putative genes and enzymatic pathways.

**Conclusions:**

The study provides useful information on *Rosa* expressed genes, with thorough annotation and an overview of expression patterns for transcripts with good accuracy.

## Background

Cultivated roses have a very ancient history and artificial crossing led to what are today perceived as the “modern rose cultivars”. Rose (genus *Rosa*) belongs to the large family of the Rosaceae (*e.g.* apple, strawberry or peach). Roses are of a high symbolic value and a great cultural importance in different societies. They are widely used as garden ornamental plants and as cut flowers. Earlier domestication of roses involved selection for a number of traits, mainly involving floral quality, such as recurrent flowering, double flowers, petal color and fragrance. Very little information is available on the molecular mechanisms that control these traits. This dearth of information limits the scope of rational selection for improvement of ornamental plants. Rose breeding practices often involve introgression of desirable traits (mainly floral) from non-elite or wild varieties with varying ploidy levels into tetraploid elite cultivars. Selection pressure has also led to the loss of important characters such as tolerance to biotic and/or abiotic stresses. In many crop species, molecular markers allow breeders to rapidly screen a large number of lines for markers associated with traits of interest, allowing the subsequent selection of relevant molecular markers and thus specific introgression of single genomic loci. However, in roses the lack of knowledge of the genetic basis upon which modern rose cultivars are established hampers molecular marker assisted selection.

The enormous progress that has been made towards understanding various aspects of plant development and resistance to biotic and abiotic stresses, as well as defining the molecular and genetic pathways associated with these processes, has mainly involved annual model species such as *Arabidopsis thaliana*, tobacco, rice or maize. Several traits such as recurrent blooming, scent production and the production of double flowers, cannot be studied using these model species, or at least only in a limited manner. The rose represents an ideal ornamental model species to address some of these characters.

During the past few years, EST sequencing using cDNA libraries has been used to identify genes expressed in *Rosa sp*[1-3]. However, these rose ESTs remained limited to genes expressed during floral development and to date sequence information for only about 5000 genes exists in the databases [4]. Despite their relatively limited number, the available ESTs for the rose have been valuable for the identification of several novel genes associated with flower characters such as the scent related germacrene D synthase, O-methyltransferases and alcohol acetyltransferase genes [3,5-10]. In recent years, *Rosa chinensis* cv. Old Blush was chosen as a model to develop tools for genomics and genetic transformation [4,11]. This diploid and recurrent flowering rose is a common ancestor of many commercial modern roses and has contributed to the recurrent flowering and the tea scent traits [12].

Here we used a combination of 454 and Illumina sequencing technologies to establish an EST database containing information on rose sequences expressed in a wide range of *R. chinensis* Old Blush tissues as well as during biotic and abiotic stresses. An *in silico* profile of gene expression and a reconstruction of rose metabolic pathways are presented and have been made accessible through web interface. Real time quantitative RT-PCR (qPCR) analyses of selected genes whose expression is specific to different organs, stress conditions and/or development stages confirmed the *in silico* data. Similarly, rose genes associated with flower development, stamen development and fragrance show consistent *in silico* expression patterns.

## Results and discussion

### RNA sampling, experimental design

**Figure 1:**
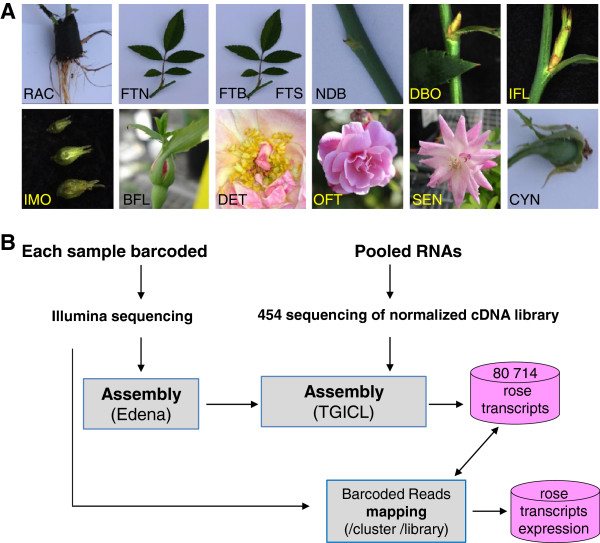
**Samples description and experimental design for next-generation sequencing. A**) 13 different tissues and conditions used for RNA purification. RAC : White young roots; FTN : Young leaves and stems; FTB : Leaves infected with *Botrytis cinerea* LR18; FTS: Leaves from water stressed plants; NDB: Dormant axillary buds (vegetative meristem); DBO: Active axillary buds (vegetative meristem); IFL: Floral bud at floral meristem transition; IMO: Floral meristem and early floral organs (sepal, petal, stamens and carpels) development; BFL: closed flower; DET: Stamens at microsporogenesis and microgametogenesis stages; OFT: open flower; SEN: senescent flower; CYN: rose hip from pollination up to early pigmentation. **B**, Sequencing and assembly strategy. Illumina reads were assembled using edena and combined with the trimmed 454 reads using TGICL to generate the final clusters assembly.

With the objective of increasing the available coding sequences for the rose, RNA samples were prepared from various organs of *R. chinensis* cv. Old Blush plants, grown under controlled greenhouse conditions, and then used to generate unidirectional cDNA libraries. To get a wide representation of genes expressed in *Rosa sp*, 13 RNA samples representing all rose plant organs and tissues were generated. Samples include vegetative and floral meristems, buds at different steps of bursting, floral organs at different developmental stages, young (white) developing roots, rose hips (cynorhodon) at early developmental stages, untreated young leaves and leaves that were subjected to biotic or abiotic stresses (Figure 1). Leaves were water stressed or infected with *Botrytis cinerea* LR18, a necrotrophic fungal pathogen known to cause severe symptoms such as grey mold in many soft fruits and ornamentals including rose, gerbera and chrysanthemum [13]. Infection by *B. cinerea* reduces the postharvest quality of rose flowers leading to substantial economic loss by growers and wholesalers [14].

### EST sequencing and clustering, sequence annotation and database creation

To obtain a reference set of transcripts, a normalized cDNA library from the above 13 different rose tissues and conditions was sequenced using Roche GS-FLX 454 Titanium technology. About 1,043,708 raw reads with an average length of 350 nucleotides corresponding to a total of 288 Mb were obtained (Table 1). In parallel, the 13 different non-normalized cDNA libraries were individually barcoded and sequenced using the Illumina technology. About 9 332 571 reads with a minimum length of 32 nucleotides corresponding to about 300 MB were obtained and assembled using edena (−overlapCutoff=22; minContigSize=80) [15].

**Table 1:** Raw data from Illumina and 454 sequencing

	**Sample**	**Number of reads**	**Number of bases**
Illumina raw data	NDB	620 667	19 861 344
	DBO	407 020	13 024 640
	IFL	746 757	23 896 224
	IMO	862 350	27 595 200
	BFL	913 850	29 243 200
	OFT	1 200 674	38 421 568
	DET	670 449	21 454 368
	SEN	726 993	23 263 776
	CYN	577 706	18 486 592
	FTN	485 234	15 527 488
	FTS	823 657	26 357 024
	FTB	451 500	14 448 000
	RAC	845 714	27 062 848
	*Total*	*9 332 571*	*298642272*
Raw 454 data		1 043 708	288 860 133

Clustering was performed using a modified version of TGICL optimized to accommodate very large datasets [16]. The input sequences were both trimmed 454 reads and 20554 contigs generated by edena using the short Illumina reads. A total of 80714 rose EST clusters longer than 100 nucleotides and based on more than 2 sequence fragments were assembled. Each fragment originated either from a 454 read or from an edena contig. These *Rosa sp.* EST sequences are available at the *ROSAseq* web interface database,http://iant.toulouse.inra.fr/R.chinensis. An additional 1248 clusters had significant matches in the *Botrytis cinerea* genome and are available as a separate set available as a tabulated file (under “*B. cinerea alignments*”) on the *ROSAseq* web interface database.

11307 rose cDNA clusters contained more than 15 reads and only 32 clusters contained over 200 reads, amongst which 3 had more than 300 reads (with a maximum of 447 reads for the most represented cluster). These figures indicate that normalization of the reference library from pooled tissue was particularly efficient (Additional file 1: Figure S1). The set of clusters that had more than 200 reads contained genes known to be highly expressed, such as genes coding for proteinase inhibitors, histones, and ribosomal proteins, but also genes with more specific expression patterns such as the floral organ identity MADS-box transcription factor *APETALA3*, and a putative terpenoid synthase coding gene whose expression is specific to mature floral tissue [17].

The clusters’ best BLASTN hits in closely related Rosaceae species with sequenced genomes (strawberry and peach, e-value1e-6) revealed that 44656 clusters had a BLASTN hit on 14252 *Fragaria vesca* transcripts with a mean nucleotide identity of 90,88%, and 36455 clusters had hits on 13033 *Prunus persica* genes with an average nucleotide identity of 85,01%. Peach, strawberry and rose have relatively small genome sizes of about 230 Mb, 240 Mb and 560 Mb respectively, and exhibit high synteny [18,19]. In the strawberry and peach genomes there are 34809 and 27852 predicted transcripts respectively, not all of them being supported by transcriptome mapping ([20]; http://www.rosaceae.org/peach/genome). Overall, about 53% and 44% of the predicted transcripts in the strawberry and peach, respectively, are represented in our *Rosa sp*. database. BLASTN (e-value 1e-6) between strawberry and peach transcripts showed that 25543 strawberry transcripts have hits on 16777 peach transcripts (65% of total peach transcripts) and 26522 peach transcripts have blast hits on 17625 strawberry transcripts (66% of total strawberry transcripts). Therefore, the observed slightly lower percentage of rose transcripts with hits in strawberry or peach transcripts can be due to the fact that some tissues or developmental stages are missing in our sampling combined with non exhaustive sequencing depth.

To have predictive peptide information, the 80714 clusters were analyzed with the FrameDP [21]. 76611 sequences were predicted to code for putative proteins which were annotated based on an automatic InterproScan analysis [22]. The OrthoMCL tool was used to generate families of proteins where each family consists of orthologs or “recent” paralogs from at least two species with a whole genome sequence [23]. Rose predicted peptides were compared to the proteomes from *F. vesca*, *P. persica* and *A. thaliana*. This method uses an all-against-all BLAST search (including within-genus and between genus BLAST) of each genus proteome, followed by a Markov cluster algorithm. The analysis is based on a BLASTp with stringent parameters, followed by a computation excluding sequences with Percent Match Cutoff lower than 80%. OrthoMCL analysis clustered 20997 putative rose peptides into 13900 protein families. 8769 OrthoMCL families corresponded to unique *Rosa sp*. genes, 4074 families corresponded to two genes and 1057 corresponded to more than two genes. The orthoMCL families that corresponded to at least two genes represent either proteins coded by different alleles or peptides from the same protein but with no overlapping amino acid sequence. Alternatively, the multiple gene families may correspond to genes subject to recent duplication events.

**Figure 2:**
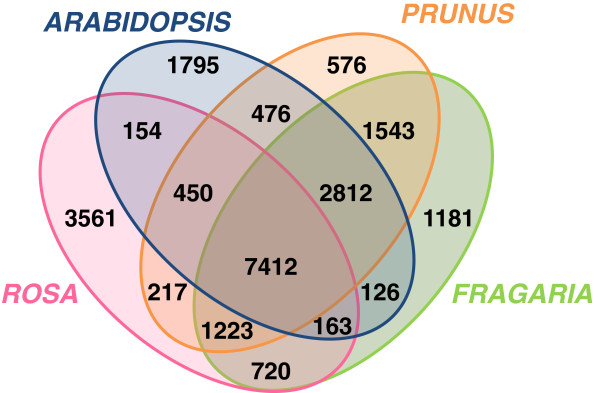
**Gene families shared between *Rosa chinensis*, *Fragaria vesc*a, *Prunus persica *and *Arabidopsis thaliana.*** OrthoMCL program was used to identify gene families shared between the four species using the following BLASTp parameters : P-value Cut-off 1e-05; Percent Identity Cut-off 0 [−F F] (i.e. low complexity filter inactivated). To increase confidence, a percent Match Cut-off 80 (i.e. Query and Match had to overlap on more than 80% of the query and match sequence length) filter was performed on the BLAST results.

The second level of OrthoMCL analysis allowed normalized inter-species comparisons (Figure 2). Common and specific OrthoMCL families (including paralogs and orthologs) were identified in the different species. The rose protein dataset contains 9518, 9302 and 8179 common families with the *F. vesca*, *P. persica* and *A. thaliana* proteomes, respectively. OrthoMCL analysis allowed the identification of 3561 gene families that appeared unique to the *Rosa* genus when compared to *F. vesca*, *P. persica* and *A. thaliana*. However, this number of gene families unique to *Rosa sp* is likely to be an overestimate since certain families may not exhibit sufficient overlap with their hit from another species. We identified 2558 peptides in the *Rosa* dataset that share a unique ortholog in the four analyzed species, *Rosa*, *Prunus*, *Fragaria* and *Arabidopsis*. Access to the protein sequences in fasta format for each OrthoMCL cluster is possible through the web portal http://iant.toulouse.inra.fr/R.chinensis.

### Gene representation in different putative pathways

Pathway Tool [24] was applied to generate a dedicated resource using the rose peptide dataset. The putative pathways identified using semi-automated tools are available at http://pathway-tools.toulouse.inra.fr/ROSACYC under *ROSAcyc*. The majority of the previously reported pathways in plants are present in the *ROSAcyc* database and can be viewed through the web portal. For example, analyses of the secondary metabolism pathways showed that the carotenoid biosynthesis superpathway is well supported in the *ROSAcyc* database by numerous putative peptides (http://pathway-tools.toulouse.inra.fr/ROSACYC/new-image?type=PATHWAY&object=CAROTENOID-PWY). The database provides information on peptides that were automatically attributed to a given metabolic pathway. Such information can be used as a basis for further data mining, such as searches of gene expression patterns.

### *In silico* expression patterns of *Rosa sp* genes

A molecular tagging approach coupled to Illumina sequencing was used to construct an “*in silico* gene expression atlas” of different rose tissues and stress conditions. The 13 different non-normalized cDNA libraries representing various rose tissues and conditions (Figure 1) were individually barcoded and sequenced using the Illumina technology (Table 1). The 9 332 571 short reads were mapped using glint software (Faraut T. and Courcelle E.; http://lipm-bioinfo.toulouse.inra.fr/download/glint/, unpublished) onto the EST clusters generated from the 454 sequencing data and counted per cluster and per library. For each cluster, total short reads counted per library are available, providing clues towards an expression pattern for the corresponding gene. RPKM normalization [25] was also performed. Raw and normalized counts are available for each cluster in the database.

**Figure 3:**
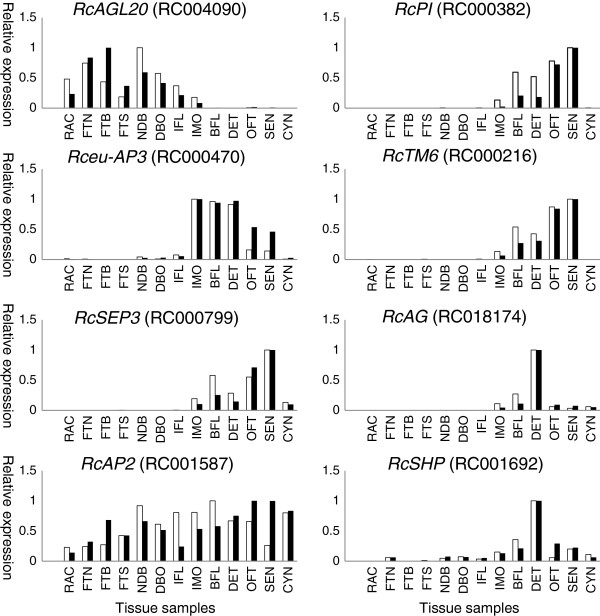
**Expression analyses by real time quantitative RT-PCR (qPCR) of eight transcripts selected *in silico*.** Relative qPCR and RPKM values were scaled with maximum expression value set to 1. White histograms: relative RPKM counts; black histograms: qPCR relative expression.

These data were first validated through a qPCR approach. Twenty-three genes whose expression was previously reported to correlate with certain physiological characters, such as color, scent biosynthesis, pollen or egg cell ploidy level, as well as developmental characters (branching, recurrent flowering, form and number of organs especially petals) in *Rosa sp* and/or in other species such as *A. thaliana*, were selected for qPCR expression profiling (Figure 3; Additional file 2: Figure S2). The correlation between RNAseq/*in silico* data and qPCR data was assessed by calculating the Pearson's product moment correlation coefficient (Additional file 3: Table S1). The statistical significance of each Pearson’s correlation coefficient was assessed using the cor.test routine in R. For most analyzed genes a high correlation coefficient was observed (mean of 0.81) and only a few genes showed low correlation between qPCR and *in silico* data (Additional file 3: Table S1). These results suggest that our *in silico* data is accurate in the different tissues and experimental conditions.

*In silico* expression can be obtained for each of the 80714 clusters, through the web portal *ROSAseq.* To address whether this atlas of gene expression allows data mining to help initiating studies of specific functions in the rose, the following three important developmental processes related to flower development and scent were analyzed.

### Rose genes involved in flower initiation and development

**Figure 4:**
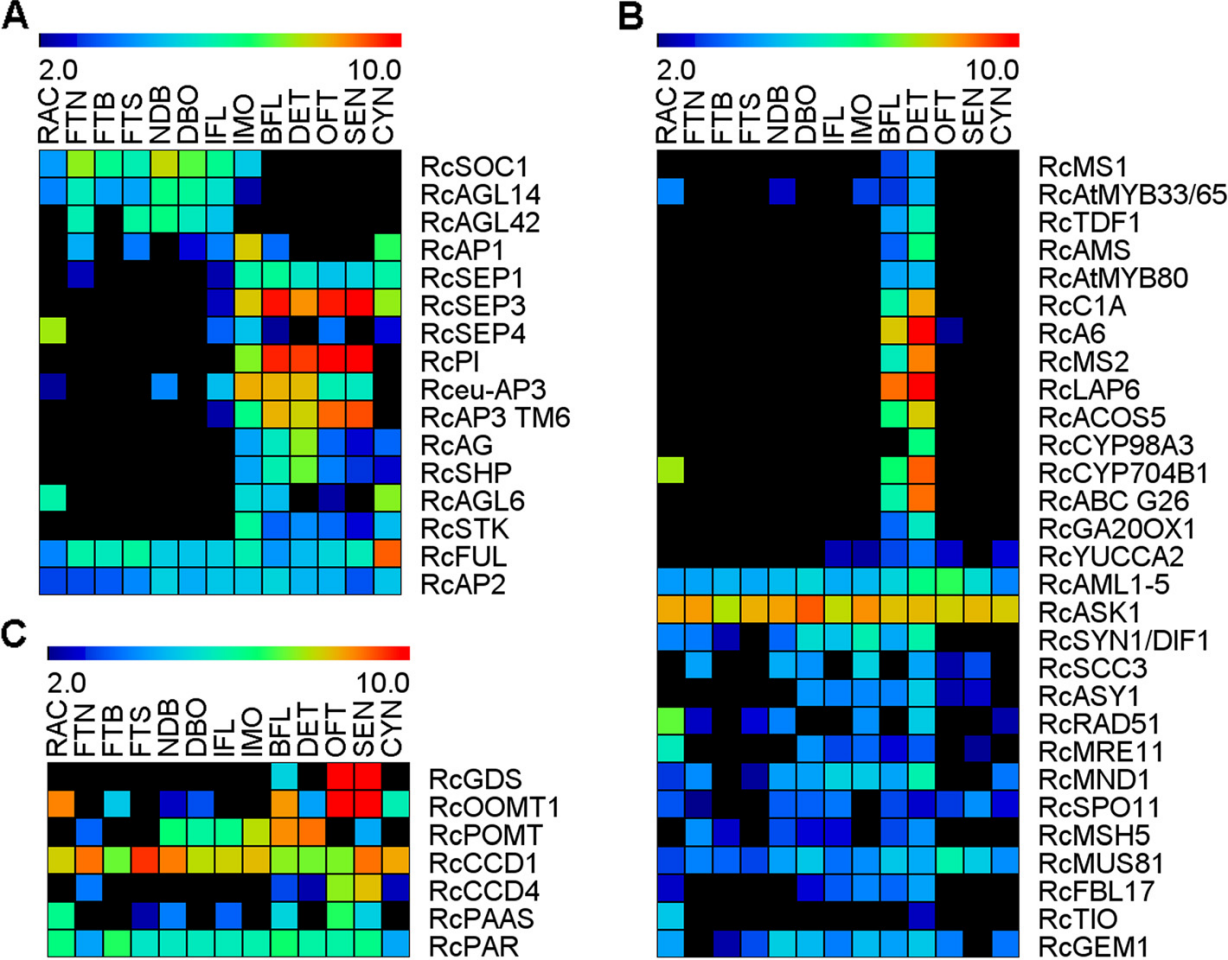
**Expression profiles of selected transcripts putatively associated with flower development and scent.** Floral development (**A**), stamen formation and meiosis (**B**) and scent biosynthesis (**C**) associated transcripts. Heatmap was generated using RPKM data (ln2 scale) using the MeV analysis tool [64].

We addressed the expression profiles for transcripts previously shown to exhibit flower specific expression patterns in *Rosa* as well as for putative orthologs of well-described *Arabidopsis* floral genes (Additional file 4: Table S2; Figure 4A). Because our dataset is fragmented due to the nature of the next generation sequencing techniques employed [26], for each *Arabidopsis* gene, more than one cluster was identified in the *ROSAseq* dataset. Interestingly, the clusters corresponding to the same gene showed similar expression patterns, thus providing another argument of the reliability of the *in silico* expression data.

Transcripts encoding the putative floral integrator *RcSOC1* (*SUPPRESSOR OF CONSTANS 1*, represented by cluster RC019456) accumulate during the vegetative phase and the floral initiation stage. No accumulation of this putative *RcSOC1* transcript was detected during later stages of floral development. This pattern of *RcSOC1* expression has already been observed in *Rosa*[27], suggesting a role of *SOC1* homologs during the vegetative phase and the floral transition. During the floral initiation process, transcripts of the ortholog of *APETALA1* (*RcAP1*) accumulate progressively with a maximum expression during early floral organogenesis (IMO), in agreement with previously reported data in *Rosa sp*. [27].

Transcripts corresponding to putative MADS box transcription factors involved in floral organ identity specification were represented in the *ROSAseq* dataset. Transcripts of the putative B class genes *RcTM6-clade* (*RCTM6*), *RcAPETALA3* (*RcAP3*) and *RcPISTILLATA* (*RcPI*) were detected at high levels in all floral samples, consistently with previously reported data [28]. Although expressed at lower levels, transcripts encoding putative orthologs of *AGAMOUS (AG)*, *SEEDSTICK* and *SHATTERPROOF (SHP)* were detected in floral samples and in the cynorhodon (rose hip). This result is consistent with previously reported data on the rose *RhAGAMOUS (RhAG)* orthologs [29,30]. Interestingly, the clusters corresponding to the putative *SHP1* did not show any particular enrichment in the hip library, but exhibited an expression pattern similar to that of *RhAG* in developing stamens. This pattern resembles the previously reported expression profile of C-function genes in *Petunia* and *Antirhinnum*, suggesting a shared C-function between *AG* and *SHP* orthologs [31]. As expected, putative homologs of the key floral developmental regulator genes *SEPALLATA1* (*SEP1*) and *SEP3* were expressed in rose floral tissues. Interestingly, *RcSEP1* and *RcSEP3* were also expressed in the cynorhodon. In agreement with this observation, the strawberry *FvSEP1/2* subfamily genes have been shown to be involved in post-fecondation receptacle tissues development and ripening [32] and *LeSEP3*-like genes are necessary for fruit ripening in tomato [33].

### Genes expressed during stamen development

In *Rosa sp* no information is available on the regulatory cascades of genes regulating anther development and meiosis. We performed a BLAST search using genes from *Arabidopsis* known to be involved in both stamen development and male gametogenesis [34-40]. Candidate clusters were readily identified in the *ROSAseq* dataset (Additional file 4: Table S2), and their *in silico* expression profile was analyzed further (Figure 4B). Orthologs of genes involved in stamen development and microsporogenesis exhibited an expression in rose stamens (DET) and flower bud (BFL) samples, and were absent from other tissues. This is the case for *MALE STERILITY1* (*MS1*)*, ABORTED MICROSPORE* (*AMS*) and *DEFECTIVE IN TAPETAL DEVELOPMENT AND FUNCTION1* (*TDF1*)*,* three genes known to be involved in tapetum development and/or microsporogenesis in *Arabidopsis*[41-44].

Orthologs of gene previously shown to be involved in early meiosis events up to microspore release in *Arabidopsis* are also represented in the rose stamens DET sample (Figure 4B). The *A6* ortholog showed specific expression in the rose stamens [45], while homologs of *AML1-5* and *ASK1*; [46,47] did not exhibit enrichment in the DET sample, thus in agreement with data reporting *AML* and ASK1 genes expression in both vegetative and reproductive tissues in *Arabidopsis*[46,48].

Similar to *Arabidopsis*, orthologs of genes involved in pollen wall formation (*ACOS5*, *CYP98A3;*[49,50]) or in sperm cell specification and division genes (*FBL17*) [51] exhibited an expression enriched in the rose stamen DET sample.

Taken together, these analyses demonstrate that this rose gene expression atlas is a reliable source for candidate genes associated with male reproductive processes.

### Scent Biosynthesis genes representation in the *ROSAseq* database

We searched the database for genes previously shown to be involved in rose scent biosynthesis (Figure 4C; Additional file 4: Table S2). Transcripts coding for the putative germacrene-D synthase (RcGDS) accumulated at high levels during flower opening and senescence (OFT and SEN samples) while low expression was observed in flower bud samples (BFL). It has been reported that the germacrene-D biosynthesis occurs during anthesis and at the onset of senescence [3]. Therefore, our *in silico* data shows that the accumulation of GDS transcripts correlates with germacrene-D biosynthesis.

Transcripts coding for two enzymes involved in the biosynthesis of the 1,3,5-trimethoxybenzene (TMB) are represented in the *ROSAseq* dataset. The transcript coding for phloroglucinol O-methyltransferase (POMT), known to catalyze the methylation of phloroglucinol to 3,5 dihydroxyanisole [52] is highly expressed in flower buds (BFL) and stamens (DET). The 3,5 dihydroxyanisole is a precursor for TMB [6,7,52]. The transcripts corresponding to the orcinol-O-methyl transferases (RcOOMT1 and RcOOMT2), known to act downstream in TMB and 3,5 dimethoxytoluene (DMT) biosynthesis, exhibited an expression during anthesis and senescence (OFT and SEN) (Figure 4C; Additional file 2: Figure S2), thus in agreement with previously reported data [6]. *RcOOMT1* and *RcOOMT2* originated from a recent gene duplication and exhibit high nucleotide identity and thus their expression cannot be discriminated [6,8].

The carotenoid cleavage oxygenases CCD1 and CCD4 have been reported to exhibit high expression levels in flowers and to be involved in the biosynthesis of terpenes, such as beta-ionone[53,54]. Similarly, our *in silico* data shows high accumulation of the putative *RcCCD1* transcripts in rose flowers (Figure 4C), but also in vegetative organs, in agreement with previously reported data [53]. Our *in silico* data shows that the putative *RcCCD4* transcripts accumulate to high levels in the flower and more specifically during flower opening and senescence (OFT and SEN), thus consistent with previously reported data [54].

2-phenylethyl alcohol is another organic volatile compound responsible for typical rose scent. Its synthesis occurs *via* two steps in *Rosa sp.* The first step is catalyzed by the phenylacetaldehyde synthase (PAAS), converting phenylalanine to phenylacetaldehyde [55]. Phenyacetaldehyde reductase (PAR) catalyzes the second step, reducing the phenylacetaldehyde to 2-phenylethyl alcohol [56]. *In silico* data showed that *RcPAAS* was expressed at low levels during late floral development. This result is not surprising, as *R. chinensis* Old Blush flowers do not produce phenylethanol [6]. According to our *in silico* data *RcPAR* expression was not restricted to flowers, thus in agreement with previous reported data [57].

Taken together these three examples show that the rose *in silico* expression atlas appears accurate and provides a valuable resource for *ab initio* gene expression analysis. For each cluster, exhaustive annotation has been performed and can be obtained through the web portal. This annotation data, combined with expression data for each cluster will allow data mining and help initiate functional studies in the rose.

It has been reported that *de novo* assemblies using RNAseq are highly complex due to allelic and splicing variants and transcriptional noise [58] but also because of sequencing errors and generation of chimeras. Furthermore, read mis-attribution between recently duplicated genes could hamper the discrimination of expression between close paralogs or alleles. This is typically the case for the *RcOOMT1* and *RcOOMT2* transcripts, which differ only by one SNP in their coding sequence [6,8], and indeed share the same *in silico* pattern (Additional file 2: Figure S2). Although our *in silico* data for most analyzed genes were either validated using qPCR or coherent with previous published data, gene expression should be validated using independent and sensitive methods, such as qPCR, before functional characterization steps.

## Conclusion

Our *de novo* sequencing and analyses permitted the generation of information on at least 20997 individual rose peptides, among which are peptides orthologous to at least 14252 different *Fragaria* proteins. The *ROSAseq* web portal provides a variety of pre-existing or specifically developed tools and pre-computed searches to conduct in-depth analyses at different levels. The navigation system provided makes it possible to (i) visualize EST cluster characteristics, (ii) explore gene function (iii) analyze gene and protein families (iv) retrieve expression patterns (v) download results of global analyses in tabulated format. The system can be consulted in a variety of ways including *via* multi-criteria queries based upon annotations, keywords, similarities (using a lucene-based retrieval system) as well as basic similarity searches (BLAST and PatScan) [59,60]. Results are presented with links allowing easy navigation through different sources of information.

The information on *Rosa sp* gene sequences in this study will also prove extremely useful to generate markers for high density genetic maps and to improve synteny studies with other Rosaceae, in particular *Fragaria*. Genetic mapping of *Rosa* has been underway for several years. However, because of the limited information on gene sequences, currently, only about 597 markers have been mapped onto the rose genetic maps, distributed over a length of 530 cM on seven linkage groups [61]. Similarly, synteny studies between *Rosa* and other Rosaceae such as strawberry has also been hampered because of the limited information on *Rosa sp* gene sequences [18,62].

Therefore, the *ROSAseq* database represents a comprehensive resource for transcript detection and accumulation, for genetic mapping and valuable prerequisite to the sequencing of the rose genome.

## Methods

### Plant material

*R. chinensis* cv. ‘Old Blush’ plants were grown in greenhouse with 16 h / 8 h day/night and 25°C/14°C day/night temperature. For floral transition samples (stage IFL), the terminal parts of growing shoot were harvested and rapidly dissected (removal of young leaves). This stage corresponds to the floral induction and floral initiation stages. Bud bursting samples were collected in the following conditions. Plants were propagated by cuttings and grown until the merging flower bud stage [63]. Bursting axillary buds (DBO) were collected from the upper part of the stem and they swelled with emerging leaf primordia. In contrast, dormant axillary buds (NDB) were harvested from the basal part of the stem and did not exhibit any growth activity. Young flowers (stages 3 to 5) were dissected from developing buds as previously described [29]. Stamens were collected after cytological analysis as previously described [39], different developmental and meiotic stages were pooled to constitute the DET sample. Developmental stages range from early events of organ specification to late ones of anther dehiscence and pollen release.

Young developing leaves and stems were harvested on seven weeks-old Rose plants, multiplied by cuttings and two successive prunings. Roses were cultivated in greenhouse at 24°C the day and 18°C the night, under a photoperiod of 16 h light/8 h dark. The control plants were watered daily for 15 min by capillarity (FTN), and the stressed plants were submitted to drought by arrest of watering for 4 days (FTS).

### RNA preparation

Total RNA was prepared as previously described [29]. Contaminating DNA was removed using the DNA-freeTM kit (Ambion, Cambridgeshire, UK).

## 454 and Illumina sequencing

RNA samples were checked for their integrity on an Agilent 2100 Bioanalyzer (Waldbroon, Germany) according to the manufacturer’s instructions. For each sample 25 μg of total RNA was pooled to generate a normalized cDNA library (GATC Biotech) and then used for 454 (Roche) sequencing. Molecular tagging of each of the 13 samples was performed by megaprimer PCR reaction (GATC Biotech) used to generate a tagged – non normalized- 3’ cDNA library for Illumina sequencing according to the manufacturers protocols (Illumina).

### RNAseq : short reads counting method

For expression analyses, Illumina reads were mapped on the 80714 rose clusters using the following BLAST parameters. A maximum of 2 mismatches was authorized and only alignments of 24 or more nucleotide were kept. Alignments were filtered keeping only the best score. However, due to dataset complexity some short reads were mapped and affected to more than one cluster. Finally, matches were counted and RPKM computed per cluster and per libraries. Heatmap was generated using RPKM data (ln2 scale) using the MeV analysis tool [64].

### qPCR analysis

One microgram total RNA (treated with DNase) was used in a reverse transcription assay and qPCR as previously described (Dubois et al., 2011). Expression levels were normalized with *Rc-alpha-TUBULIN*, *RcTCTP, RcACTIN* and *RcEF1-alpha* reference genes [4]. At least two independent biological replicates were used for each experiment and two qPCR technical replicates were performed for each biological replicate, with similar results. Primer sequences are available as Additional file 5: Table S3. The correlation between the RNAseq results and qPCR data was assessed by calculating the Pearson's product moment correlation coefficient [65,66].

### WEB portal

*ROSAseq* Web portal: http://iant.toulouse.inra.fr/R.chinensis

Login: guest

Password: guest

*ROSACYC* pathway tool portal: http://pathway-tools.toulouse.inra.fr/ROSACYC

Login: guest

Password: guest

## Abbreviations

RAC: White young roots; FTN: Young leaves and stems; FTB: Leaves infected with *Botrytis cinerea* LR18; FTS: Leaves from water stressed plants; NDB: Dormant axillary buds (vegetative meristem); DBO: Active axillary buds (vegetative meristem); IFL: Floral bud at floral meristem transition; IMO: Floral meristem and early floral organs (sepal, petal, stamens and carpels) development; BFL: Closed flower; OFT: Open flower; DET: Stamens at microsporogenesis and microgametogenesis stages; SEN: Senescent flower; CYN: Rose hip (cynorhodon) from pollination up to early pigmentation; EST: Expressed sequence tag; qPCR: Real time quantitative RT-PCR; SOC1: Supressor of constans1; AG: Agamous; SHP: Shatterproof; SEP: SEPALLATA; MS1: Male sterility1; AMS: Aborted microspore; TDF1: Defective in tapetal development and function1; RcGDS: Germacrene-D synthase; TMB: 1,3,5-trimethoxybenzene; POMT: Phloroglucinol O-methyltransferase; OOMT: Orcinol-O-methyl transferase; DMT: 3,5 dimethoxytoluene; PAAS: Phenylacetaldehyde synthase; PAR: Phenyacetaldehyde reductase.

## Competing interests

The authors declare no competing interests.

## Authors’ contributions

AD, SC, OR, BP, LC: carried out the molecular genetic studies and participated to the sequence analyses. MB, AD, OR, JG FF, J-PO, SS, RA, SB, ML conceived experiments and prepared material. MB, AD, SC, JG: designed and coordinated experiments. SC, LC, JG: analyzed sequence data and designed the *ROSAseq* and *ROSACYC* web interface. MB and AD wrote the paper. All authors read and approved the final manuscript.

## Supplementary Material

Additional file 1 **Figure S1.** Reads distribution among the 80714 rose clusters. Click here for file

Additional file 2 **Figure S2.** Expression analyses of selected transcripts *in silico* (RPKM values) and using qPCR. Click here for file

Additional file 3 **Table S1. ***In silico* and qPCR Pearson correlation. Click here for file

Additional file 4 **Table S2.** Genes used for rose *in silico* expression validation. Click here for file

Additional file 5 **Table S3.** Primers used in qPCR validation. Click here for file
