# Supplementary material for: Transcriptome database resource and gene expression atlas for the rose
Source: BMC Genomics. 2012 Nov 20;13:638. doi: 10.1186/1471-2164-13-638 (PMC3518227; doi:10.1186/1471-2164-13-638)
Supplement: Additional file 1 — Figure S1. Reads distribution among the 80714 rose clusters. [file 1471-2164-13-638-S1.pptx]

## Slide 1
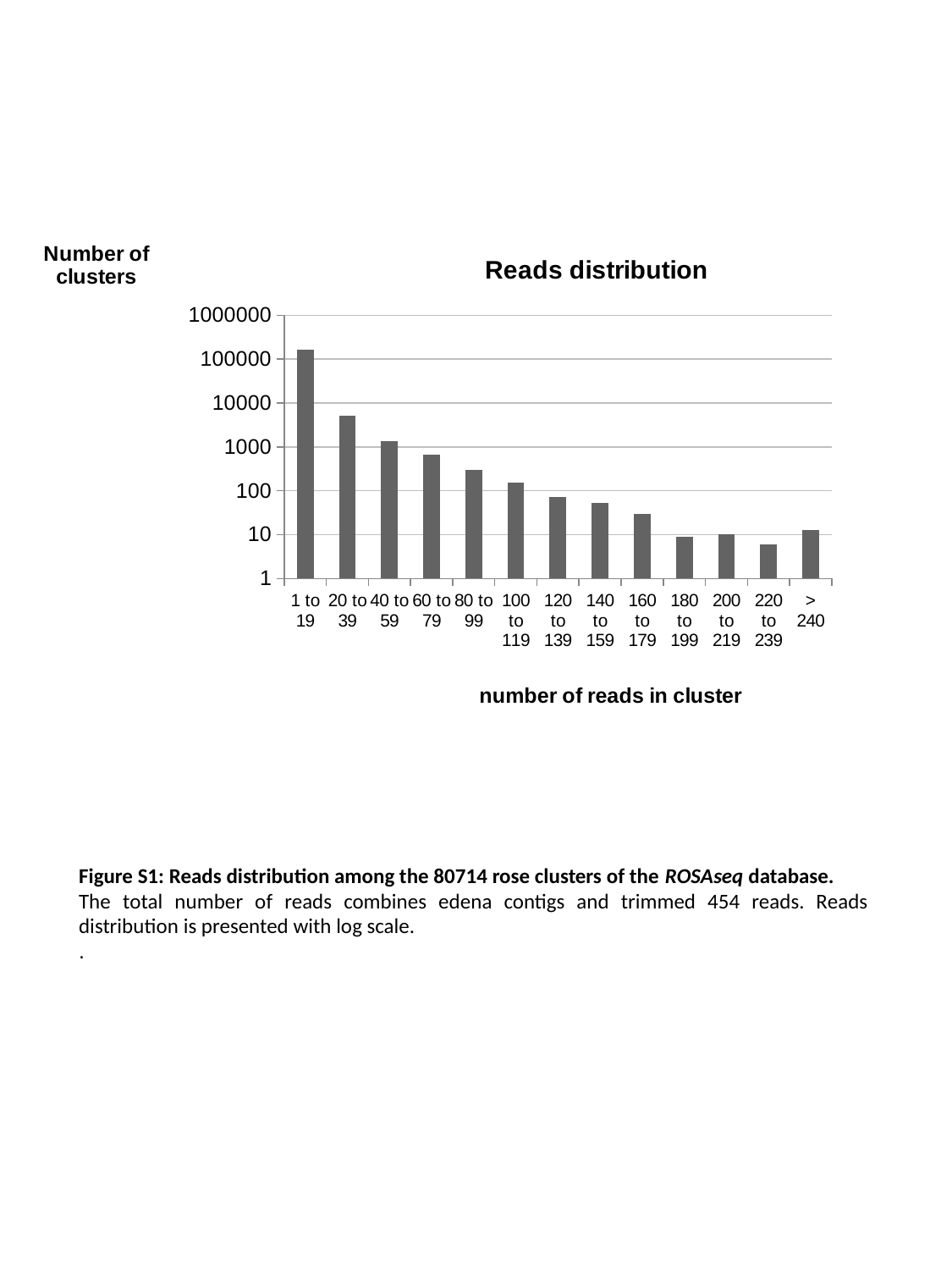

### Chart: Reads distribution
| Category | |
|---|---|
| 1 to 19 | 160290.0 |
| 20 to 39 | 5083.0 |
| 40 to 59 | 1371.0 |
| 60 to 79 | 656.0 |
| 80 to 99 | 293.0 |
| 100 to 119 | 152.0 |
| 120 to 139 | 72.0 |
| 140 to 159 | 52.0 |
| 160 to 179 | 30.0 |
| 180 to 199 | 9.0 |
| 200 to 219 | 10.0 |
| 220 to 239 | 6.0 |
| > 240 | 13.0 |Figure S1: Reads distribution among the 80714 rose clusters of the ROSAseq database.
The total number of reads combines edena contigs and trimmed 454 reads. Reads distribution is presented with log scale.
.
