# Supplementary material for: Transcriptome database resource and gene expression atlas for the rose
Source: BMC Genomics. 2012 Nov 20;13:638. doi: 10.1186/1471-2164-13-638 (PMC3518227; doi:10.1186/1471-2164-13-638)
Supplement: Additional file 2 — Figure S2. Expression analyses of selected transcripts in silico (RPKM values) and using qPCR. [file 1471-2164-13-638-S2.pptx]

## Slide 1
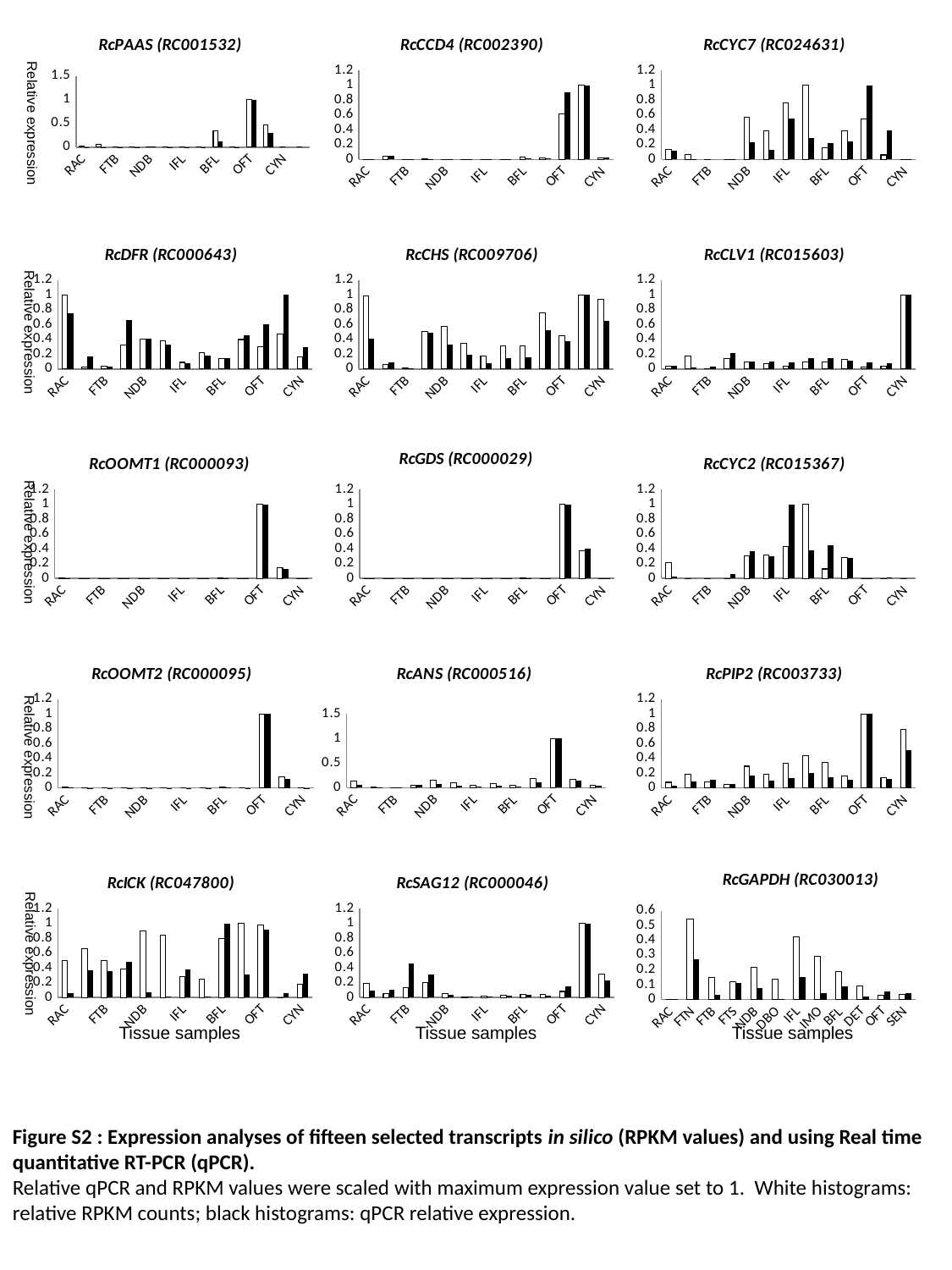

### Chart: RcPAAS (RC001532)
| Category | relative RPKM counts | relative QPCR measure |
|---|---|---|
| RAC | 0.029057289087006805 | 0.001997579427480351 |
| FTN | 0.050602608552088514 | 0.0037942246272980817 |
| FTB | 0.0 | 0.00013201603024369103 |
| FTS | 0.0 | 0.00015771641611818504 |
| NDB | 0.0 | 0.00506881814809824 |
| DBO | 0.0 | 0.002465293227089491 |
| IFL | 0.0 | 0.003556543551663702 |
| IMO | 0.0 | 0.00027710054915213307 |
| BFL | 0.34200099058940114 | 0.111285888650354 |
| DET | 0.0 | 0.0010281143817883302 |
| OFT | 1.0 | 1.0 |
| SEN | 0.464421330691762 | 0.29983635626848404 |
| CYN | 0.0 | 0.0 |
### Chart: RcCCD4 (RC002390)
| Category | relative RPKM counts | relative QPCR measure |
|---|---|---|
| RAC | 0.0 | 3.5920628911289007e-06 |
| FTN | 0.04331332057795082 | 0.043744172422574404 |
| FTB | 0.0 | 0.008546198546260002 |
| FTS | 0.006366695256317211 | 0.00229541837571645 |
| NDB | 0.0 | 0.004189595624267351 |
| DBO | 0.0 | 0.004362721592479632 |
| IFL | 0.0 | 0.00315242018887639 |
| IMO | 0.0 | 0.002217340209266741 |
| BFL | 0.0281388137494227 | 0.012066221406699901 |
| DET | 0.01642805304479781 | 0.011906234487592501 |
| OFT | 0.6131490400475041 | 0.9018685717131399 |
| SEN | 1.0 | 1.0 |
| CYN | 0.018011479844296404 | 0.019849528413650497 |
### Chart: RcCYC7 (RC024631)
| Category | relative RPKM counts | relative QPCR measure |
|---|---|---|
| RAC | 0.13868803902779003 | 0.11462897923114002 |
| FTN | 0.0605017858698493 | 0.0012171032495690904 |
| FTB | 0.0 | 0.0 |
| FTS | 0.0 | 0.0027309990049624706 |
| NDB | 0.5693004617126919 | 0.225735856670672 |
| DBO | 0.38457182681418206 | 0.12677457110175697 |
| IFL | 0.760388535586723 | 0.5499161561099649 |
| IMO | 1.0 | 0.28782841133144915 |
| BFL | 0.15715654673752102 | 0.222861618775075 |
| DET | 0.38988587856085116 | 0.24147966040076502 |
| OFT | 0.5409443331300651 | 1.0 |
| SEN | 0.059456398640996715 | 0.39078145997023406 |
| CYN | 0.0 | 0.00014389258419702307 |Relative expression
### Chart: RcDFR (RC000643)
| Category | relative RPKM counts | relative QPCR measure |
|---|---|---|
| RAC | 1.0 | 0.75044328783865 |
| FTN | 0.028006082769108202 | 0.170365468193085 |
| FTB | 0.031771606502769806 | 0.027580068176113505 |
| FTS | 0.32417900720518505 | 0.6640198689887271 |
| NDB | 0.3995437923168831 | 0.413291119424838 |
| DBO | 0.3765523733661611 | 0.33177466079440615 |
| IFL | 0.08803722075382904 | 0.07569781092120502 |
| IMO | 0.222021072450125 | 0.17752638239192506 |
| BFL | 0.14138817480719804 | 0.148632589034138 |
| DET | 0.39749809913465517 | 0.454943511006953 |
| OFT | 0.30269017705202905 | 0.6030107707830981 |
| SEN | 0.4718671928744701 | 1.0 |
| CYN | 0.15972699952931005 | 0.293560410611038 |
### Chart: RcCHS (RC009706)
| Category | relative RPKM counts | relative QPCR measure |
|---|---|---|
| RAC | 0.9829850194192711 | 0.4082727763356771 |
| FTN | 0.06527032858640032 | 0.0855104298100581 |
| FTB | 0.008230072128722024 | 0.009859120232296362 |
| FTS | 0.5002003575611871 | 0.4873308361776891 |
| NDB | 0.572714382590469 | 0.3329420659563281 |
| DBO | 0.3504099623944281 | 0.19514625953534503 |
| IFL | 0.175482399358856 | 0.07500534201101702 |
| IMO | 0.3088588866284461 | 0.145834925590537 |
| BFL | 0.31462301954256805 | 0.15422194747227705 |
| DET | 0.7615745021885222 | 0.5202004361147712 |
| OFT | 0.44584180999938305 | 0.37628796848848206 |
| SEN | 1.0 | 1.0 |
| CYN | 0.940678749768818 | 0.6480169134934401 |
### Chart: RcCLV1 (RC015603)
| Category | relative RPKM counts | relative QPCR measure |
|---|---|---|
| RAC | 0.0328617478510029 | 0.04854021140970171 |
| FTN | 0.17183022922636101 | 0.017190441973434206 |
| FTB | 0.0 | 0.0335862111874762 |
| FTS | 0.13449140401146106 | 0.21390553536774004 |
| NDB | 0.09375000000000011 | 0.0970291081443912 |
| DBO | 0.07279727793696293 | 0.10337767657291601 |
| IFL | 0.0359957020057307 | 0.09202848870833541 |
| IMO | 0.09625716332378231 | 0.14746921653758605 |
| BFL | 0.08927292263610313 | 0.14409724948659206 |
| DET | 0.130282951289398 | 0.105859380250535 |
| OFT | 0.021310888252149003 | 0.08509936583988431 |
| SEN | 0.03751790830945561 | 0.07372122588234183 |
| CYN | 1.0 | 1.0 |Relative expression
### Chart: RcOOMT1 (RC000093)
| Category | relative RPKM counts | relative QPCR measure |
|---|---|---|
| RAC | 0.010996249729005404 | 0.000732367058154936 |
| FTN | 0.0 | 1.9086564694274207e-05 |
| FTB | 0.0007207720672988741 | 0.0002998660377723691 |
| FTS | 6.793258340993143e-05 | 5.581141161370092e-05 |
| NDB | 0.00014199626640559602 | 0.00015057496639808804 |
| DBO | 0.00022047434110299003 | 0.00022113744263640306 |
| IFL | 3.629610954754461e-05 | 2.865984425305971e-05 |
| IMO | 3.2372205812675016e-05 | 0.00014282389565168597 |
| BFL | 0.009818588120577638 | 0.0021217448167204006 |
| DET | 0.00048190442743868217 | 0.0011562240610206603 |
| OFT | 1.0 | 1.0 |
| SEN | 0.14756305958545904 | 0.12524502975602803 |
| CYN | 0.0013454084930934404 | 0.00015285948521893207 |
### Chart: RcGDS (RC000029)
| Category | relative RPKM counts | relative QPCR measure |
|---|---|---|
| RAC | 0.0 | 1.3079535956834704e-07 |
| FTN | 0.0 | 0.00014679378213840603 |
| FTB | 0.0 | 0.002573150462137811 |
| FTS | 0.0 | 2.0641810139871913e-05 |
| NDB | 0.000277256941996908 | 0.00021706678911511003 |
| DBO | 0.00042998322360537406 | 0.0002850745975449812 |
| IFL | 0.00042528395340203705 | 6.9828176689273e-05 |
| IMO | 0.0 | 0.000190328244974596 |
| BFL | 0.008070996574232032 | 0.007619446140201071 |
| DET | 0.00025611022608189 | 0.000863606778219492 |
| OFT | 1.0 | 1.0 |
| SEN | 0.3783029995441721 | 0.39943492077801507 |
| CYN | 0.000561562789298822 | 8.164222270836111e-05 |
### Chart: RcCYC2 (RC015367)
| Category | relative RPKM counts | relative QPCR measure |
|---|---|---|
| RAC | 0.21163743304274804 | 0.023804255459326802 |
| FTN | 0.0 | 0.0 |
| FTB | 0.0 | 0.0 |
| FTS | 0.0 | 0.056078855379841704 |
| NDB | 0.30217414137170506 | 0.3699299207905751 |
| DBO | 0.312992332738158 | 0.29451071060155304 |
| IFL | 0.42548051675244314 | 1.0 |
| IMO | 1.0 | 0.3766895776360031 |
| BFL | 0.12792773868291102 | 0.4448742631036011 |
| DET | 0.27990757273395706 | 0.27328396194358007 |
| OFT | 0.0 | 0.00433835854102907 |
| SEN | 0.0 | 0.008437883708002632 |
| CYN | 0.0 | 0.0 |Relative expression
### Chart: RcOOMT2 (RC000095)
| Category | relative RPKM counts | relative QPCR measure |
|---|---|---|
| RAC | 0.014749456828199602 | 0.004542252399056972 |
| FTN | 0.0 | 1.3459838143571802e-05 |
| FTB | 0.00034207181638434814 | 0.00106004827292078 |
| FTS | 5.9065816742142116e-05 | 4.744088188645431e-05 |
| NDB | 0.0002468866153034861 | 9.767789389185883e-05 |
| DBO | 0.0002558102279048161 | 0.00019371692898046102 |
| IFL | 6.331515607610911e-05 | 4.241951414271512e-05 |
| IMO | 0.0 | 0.00015160505235055 |
| BFL | 0.0100284408281622 | 0.004448328748540052 |
| DET | 7.606317407801033e-05 | 0.0010211804894674399 |
| OFT | 1.0 | 1.0 |
| SEN | 0.148100949004953 | 0.127020114772359 |
| CYN | 0.0016716900939826398 | 7.98413834914699e-05 |
### Chart: RcANS (RC000516)
| Category | relative RPKM counts | relative QPCR measure |
|---|---|---|
| RAC | 0.13222546956200804 | 0.05282387383427441 |
| FTN | 0.012089413783128703 | 0.010062979962438903 |
| FTB | 0.0 | 0.0010267462649843403 |
| FTS | 0.0455347969636819 | 0.05220351056028551 |
| NDB | 0.147581618505182 | 0.07353232610828701 |
| DBO | 0.09735868253319101 | 0.04164136695923501 |
| IFL | 0.05065683819935562 | 0.018816322505931297 |
| IMO | 0.0818628868961527 | 0.04284114346634692 |
| BFL | 0.05443727369754531 | 0.028247729530375198 |
| DET | 0.19097184124165897 | 0.10957962443335202 |
| OFT | 1.0 | 1.0 |
| SEN | 0.171585887704109 | 0.15271513519278906 |
| CYN | 0.05360936829820543 | 0.04474030523663151 |
### Chart: RcPIP2 (RC003733)
| Category | relative RPKM counts | relative QPCR measure |
|---|---|---|
| RAC | 0.07665372631148802 | 0.0336230884214313 |
| FTN | 0.18724779077488904 | 0.08936187163087621 |
| FTB | 0.08165960055166792 | 0.10836737885120501 |
| FTS | 0.0507304489962711 | 0.051752056929953306 |
| NDB | 0.292999438116157 | 0.16240888429699205 |
| DBO | 0.18402206671093602 | 0.10166803189894699 |
| IFL | 0.33021402666394206 | 0.13210598721884298 |
| IMO | 0.4347065433927581 | 0.19646893501618903 |
| BFL | 0.34460846912192905 | 0.14229475458709007 |
| DET | 0.161738264289728 | 0.11180630147693602 |
| OFT | 1.0 | 1.0 |
| SEN | 0.141081370996578 | 0.12134990395152001 |
| CYN | 0.7949992337947591 | 0.51183455707277 |Relative expression
### Chart: RcICK (RC047800)
| Category | relative RPKM counts | relative QPCR measure |
|---|---|---|
| RAC | 0.5038098140810742 | 0.05671523175330521 |
| FTN | 0.6592502285888462 | 0.3639485916624741 |
| FTB | 0.4983236818043291 | 0.3514642197766421 |
| FTS | 0.38707711063700107 | 0.47662196724760214 |
| NDB | 0.899420908259677 | 0.06558197089634339 |
| DBO | 0.83846388296251 | 0.011053952991773704 |
| IFL | 0.2761353245961599 | 0.3804006357551051 |
| IMO | 0.24626638220054903 | 0.015537350254537202 |
| BFL | 0.7994513867723249 | 1.0 |
| DET | 1.0 | 0.3063761095868591 |
| OFT | 0.982627247790308 | 0.9164383453336309 |
| SEN | 0.0 | 0.0633995081831135 |
| CYN | 0.18287107589149604 | 0.3159021413805881 |
### Chart: RcSAG12 (RC000046)
| Category | relative RPKM counts | relative QPCR measure |
|---|---|---|
| RAC | 0.19030236788092203 | 0.09787087562946019 |
| FTN | 0.05653962320823672 | 0.10253308795493903 |
| FTB | 0.13754234318420705 | 0.45472430042011097 |
| FTS | 0.197926226909406 | 0.3052210118554491 |
| NDB | 0.05496270462395911 | 0.040237439148042016 |
| DBO | 0.011328366178850902 | 0.0159257893963309 |
| IFL | 0.013998281242178001 | 0.011032530410992905 |
| IMO | 0.025731306422814505 | 0.02050503664702561 |
| BFL | 0.043619737347105725 | 0.0314744254204887 |
| DET | 0.03975253224756792 | 0.0258176675754019 |
| OFT | 0.08145743988519359 | 0.14540671226190302 |
| SEN | 1.0 | 1.0 |
| CYN | 0.3196785255394071 | 0.23520036721772103 |
### Chart: RcGAPDH (RC030013)
| Category | relative RPKM counts | relative QPCR measure |
|---|---|---|
| RAC | 0.0 | 2.0641202546663718e-05 |
| FTN | 0.5439077225009731 | 0.27068397349128803 |
| FTB | 0.14939549351058104 | 0.0297340794589867 |
| FTS | 0.11897576419618201 | 0.11097992457870699 |
| NDB | 0.21564125826220604 | 0.07856017285663185 |
| DBO | 0.13745348169817304 | 0.00232230574561012 |
| IFL | 0.4246542370998511 | 0.150692021252563 |
| IMO | 0.289644702005147 | 0.04265182746852232 |
| BFL | 0.18781359352724405 | 0.08874511644554273 |
| DET | 0.09222195478698784 | 0.01763305596717681 |
| OFT | 0.027698061505989503 | 0.05565413558854511 |
| SEN | 0.035400196256318205 | 0.040075522667600005 |Relative expression
Tissue samples
Tissue samples
Tissue samples
Figure S2 : Expression analyses of fifteen selected transcripts in silico (RPKM values) and using Real time quantitative RT-PCR (qPCR).
Relative qPCR and RPKM values were scaled with maximum expression value set to 1. White histograms: relative RPKM counts; black histograms: qPCR relative expression.
