# Supplementary material for: Transcriptome database resource and gene expression atlas for the rose
Source: BMC Genomics. 2012 Nov 20;13:638. doi: 10.1186/1471-2164-13-638 (PMC3518227; doi:10.1186/1471-2164-13-638)
Supplement: Additional file 3 — Table S1. In silico and qPCR Pearson correlation. [file 1471-2164-13-638-S3.docx]

|  |  |  | |
| --- | --- | --- | --- |
|  | MAX count | pearson | p-value |
| *GAPDH (RC030013)* | 540,11 | 0,9203865 | 4,12E-06 |
| *AG (RC018174)* | 182,86 | 0,9829545 | 9,68E-10 |
| *SHP (RC0a01692)* | 139,13 | 0,953677 | 2,24E-07 |
| *AP2 (RC001587)* | 35,93 | 0,1598035 | 0,301 |
| *AP3 (TM6 clade) (RC000216)* | 614,61 | 0,9771323 | 4,82E-09 |
| *Eu-AP3 (RC000470)* | 345,74 | 0,9483537 | 4,02E-07 |
| *PI (RC000382)* | 1316,6 | 0,9267774 | 2,63E-06 |
| *SEP3 (RC000799)* | 1461,12 | 0,9395234 | 9,42E-07 |
| *DIF1 (RC001060)* | 36034,03 | 0,9883679 | 1,20E-10 |
| *PAAS (RC001532)* | 121,14 | 0,9681512 | 2,93E-08 |
| *OOMT1 (RC000093)* | 40775,72 | 0,9997085 | < 2,2e-16 |
| *OOMT2 (RC000095)* | 23533,07 | 0,9997386 | < 2,2e-16 |
| *CHS (RC009706)* | 648,84 | 0,0149553 | 0,4807 |
| *CCD4 (RC002390)* | 303,14 | 0,9795915 | 2,59E-09 |
| *GDS (RC000029)* | 4255,98 | 0,99981 | < 2,2e-16 |
| *DFR (RC000643)* | 552,38 | 0,7364547 | 0,002047 |
| *ANS (RC000516)* | 2005,06 | 0,9928312 | 8,42E-12 |
| *AGL20 (RC004090)* | 297,89 | 0,7592067 | 0,001307 |
| *CLV1 (RC015603)* | 111,68 | 0,973592 | 1,06E-08 |
| *ICK (RC047800)* | 32,81 | 0,354834 | 0,1171 |
| *PIP2 (RC003733)* | 3915,4 | 0,935795 | 1,30E-06 |
| *CYC2 (RC015367)* | 95,21 | 0,5663638 | 0,0218 |
| *CYC7 (RC024631)* | 229,58 | 0,5467237 | 0,02659 |
| *SAG12 (RC000046)* | 4794,16 | 0,9292601 | 2,19E-06 |

**Table S1**: Pearson correlation between expressions of selected genes as measured by qPCR and *in silico* analyses. The statistical significance of each Pearson’s correlation coefficient was assessed using the stringent cor.test routine in R, generating the p-value.
