# Supplementary material for: Transcriptome database resource and gene expression atlas for the rose
Source: BMC Genomics. 2012 Nov 20;13:638. doi: 10.1186/1471-2164-13-638 (PMC3518227; doi:10.1186/1471-2164-13-638)
Supplement: Additional file 4 — Table S2. Genes used for rose in silico expression validation. [file 1471-2164-13-638-S4.docx]

| Cluster reference | *Rosa chinensis* putative gene | Accession | Identity (%) | e-value for the Blastp search against Arabidopsis protein database (TAIR) | Reference |
| --- | --- | --- | --- | --- | --- |
| RC019456 | RcSOC1 | AT2G45660 | 76 | 1.07251e-32 | [[1](#_ENREF_1)] |
| RC012786 | RcAP1 | AT1G69120 | 49 | 5.15872e-21 | [[1](#_ENREF_1)] |
| RC001958 | RcSEP1 | AT5G15800 | 68 | 4.94646e-91 | [[2](#_ENREF_2)] |
| RC000799 | RcSEP3 | AT1G24260 | 71 | 3.64192e-64 | [[2](#_ENREF_2)] |
| RC040855 | RcSEP4 | AT2G03710 | 65 | 1.39984e-41 | [[2](#_ENREF_2)] |
| RC000382 | RcPI | AT5G20240 | 56 | 5.76273e-63 | [[3](#_ENREF_3)] |
| RC000470 | RcEu-AP3 | AT3G54340 | 51 | 1.68004e-61 | [[3](#_ENREF_3)] |
| RC000216 | RcAP3 TM6 | AT3G54340 | 54 | 1.33747e-57 | [[3](#_ENREF_3)] |
| RC018174 | RcAG | AT4G18960 | 58 | 1.70362e-60 | [[4](#_ENREF_4)] |
| RC006175 | RcSHP | AT3G58780 | 65 | 5.74377e-74 | [[4](#_ENREF_4)] |
| RC045163 | RcAGL6 | AT2G45650 | 67 | 1.84801e-48 | [[5](#_ENREF_5)] |
| RC023413 | RcFUL | AT5G60910 | 76 | 7.08588e-30 | [[6](#_ENREF_6)] |
| RC001587 | RcAP2 | AT4G36920 | 83 | 9.99443e-57 | [[7](#_ENREF_7)] |
| RC022942 | RcMS1 | At5g22260 | 59 | 2.9E-75 | [[8](#_ENREF_8)] |
| RC038621 | RcAtMYB33/65 | At5g06100 | 70 | 7.9E-38 | [[9](#_ENREF_9)] |
| RC002712 | RcTDF1 | At3g28470 | 71 | 7.2E-43 | [[10](#_ENREF_10)] |
| RC005063 | RcAMS | At2g16910 | 70 | 1.1E-32 | [[11](#_ENREF_11)] |
| RC013391 | RcAtMYB80 | At5g56110 | 89 | 3.1E-64 | [[12](#_ENREF_12)] |
| RC019442 | RcC1A | At3g49340 | 40 | 4.3E-25 | [[13](#_ENREF_13)] |
| RC000427 | RcA6 | At4g14080 | 58 | 8.0E-124 | [[14](#_ENREF_14)] |
| RC001400 | RcMS2 | At3g11980 | 67 | 1.8E-127 | [[15](#_ENREF_15)] |
| RC000986 | RcLAP6 | At1g02050 | 81 | 1.7E-97 | [[16](#_ENREF_16)] |
| RC002297 | RcACOS5 | At1g62940 | 65 | 2.5E-106 | [[17](#_ENREF_17)] |
| RC008618 | RcCYP98A3 | At2g40890 | 63 | 1.1E-56 | [[18](#_ENREF_18)] |
| RC043565 | RcCYP704B1 | At1g69500 | 84 | 2.7E-32 | [[19](#_ENREF_19)] |
| RC002398 | RcABC G26 | At3g13220 | 67 | 9.4E-109 | [[20](#_ENREF_20)] |
| RC062316 | RcGA20OX1 | At4g25420 | 68 | 8.8E-52 | [[21](#_ENREF_21)] |
| RC040611 | RcYUCCA2 | At4g13260 | 76 | 1.7E-22 | [[22](#_ENREF_22)] |
| RC000041 | RcAML1-5 | AT1G29400 | 78 | 1,00E-67 | [[23](#_ENREF_23)] |
| RC000924 | RcASK1 | At1g75950 | 77 | 2.5E-69 | [[24](#_ENREF_24)] |
| RC026205 | RcSYN1/DIF1 | At5g05490 | 45 | 8.4E-33 | [[25](#_ENREF_25)] |
| RC027610 | RcSCC3 | At2g47980 | 71 | 1.8E-63 | [[26](#_ENREF_26)] |
| RC005885 | RcASY1 | AT1G67370 | 55 | 6.6E-60 | [[27](#_ENREF_27)] |
| RC014005 | RcRAD51 | At5g20850 | 81 | 9.1E-40 | [[28](#_ENREF_28)] |
| RC037453 | RcMRE11 | At5g54260 | 43 | 1.8E-9 | [[29](#_ENREF_29)] |
| RC025245 | RcMND1 | At4g29170 | 76 | 3.4E-35 | [[30](#_ENREF_30)] |
| RC007406 | RcSPO11 | At3g13170 | 46 | 5.8E-44 | [[31](#_ENREF_31)] |
| RC004253 | RcMSH5 | At3g20475 | 55 | 1.1E-22 | [[32](#_ENREF_32)] |
| RC004976 | RcMUS81 | At4g30870 | 51 | 3,00E-08 | [[33](#_ENREF_33)] |
| RC004996 | RcFBL17 | At3g54650 | 61 | 2,00E-56 | [[34](#_ENREF_34)] |
| RC039419 | RcTIO | At1g50240 | 57 | 3.4E-34 | [[35](#_ENREF_35)] |
| RC008332 | RcGEM1 | At2g35630 | 66 | 9.7E-125 | [[36](#_ENREF_36)] |
| RC000029 | RcGDS | BQ105086 | 100 | 4.29293e-142 | [[37](#_ENREF_37)] |
| RC000093 | RcOOMT1 | AJ439741 | 99 | 0.00000 | [[38](#_ENREF_38)] |
| RC075233 | RcPOMT | AB121046 | 99 | 0.00000 | [[39](#_ENREF_39)] |
| RC047669 | RcCCD1 | GQ150681 | 96 | 9.52653e-48 | [[40](#_ENREF_40)] |
| RC002390 | RcCCD4 | AT4G19170 | 68 | 1.28688e-107 | [[41](#_ENREF_41)] |
| RC050826 | RcPAAS | DQ192639 | 97 | 4.83355e-87 | [[42](#_ENREF_42)] |
| RC002888 | RcPAR | AB426519 | 99 | 1.03796e-153 | [[43](#_ENREF_43)] |

**Table S2 :** List of selected genes used for rose *in silico* expression validation.

References

1. Remay A, Lalanne D, Thouroude T, Le Couviour F, Hibrand-Saint Oyant L, Foucher F: **A survey of flowering genes reveals the role of gibberellins in floral control in rose**. *Theor Appl Genet* 2009, **119**(5):767-781.

2. Pelaz S, Ditta GS, Baumann E, Wisman E, Yanofsky MF: **B and C floral organ identity functions require SEPALLATA MADS-box genes**. *Nature* 2000, **405**(6783):200-203.

3. Hibino Y, Kitahara K, Hirai S, Matsumoto S: **Structural and functional analysis of rose class B MADS-box genes ‘MASAKO BP, euB3, and B3’: Paleo-type AP3 homologue ‘MASAKO B3’ association with petal development**. *Plant Sci* 2006, **170**:778-785.

4. Kitahara K, Hibino Y, Aida R, Matsumoto S: **Ectopic expression of the rose AGAMOUS-like MADS-box genes 'MASAKO C1 and D1' causes similar homeotic transformation of sepal and petal in Arabidopsis and sepal in Torenia**. *Plant Sci* 2004, **166**(5):1245-1252.

5. Schauer SE, Schluter PM, Baskar R, Gheyselinck J, Bolanos A, Curtis MD, Grossniklaus U: **Intronic regulatory elements determine the divergent expression patterns of AGAMOUS-LIKE6 subfamily members in Arabidopsis**. *Plant J* 2009, **59**(6):987-1000.

6. Gu Q, Ferrandiz C, Yanofsky MF, Martienssen R: **The FRUITFULL MADS-box gene mediates cell differentiation during Arabidopsis fruit development**. *Development* 1998, **125**(8):1509-1517.

7. Wollmann H, Mica E, Todesco M, Long JA, Weigel D: **On reconciling the interactions between APETALA2, miR172 and AGAMOUS with the ABC model of flower development**. *Development* 2010, **137**(21):3633-3642.

8. Wilson ZA, Morroll SM, Dawson J, Swarup R, Tighe PJ: **The Arabidopsis MALE STERILITY1 (MS1) gene is a transcriptional regulator of male gametogenesis, with homology to the PHD-finger family of transcription factors**. *Plant J* 2001, **28**(1):27-39.

9. Millar AA, Gubler F: **The Arabidopsis GAMYB-like genes, MYB33 and MYB65, are MicroRNA-regulated genes that redundantly facilitate anther development**. *Plant Cell* 2005, **17**(3):705-721.

10. Zhu J, Chen H, Li H, Gao JF, Jiang H, Wang C, Guan YF, Yang ZN: **Defective in Tapetal development and function 1 is essential for anther development and tapetal function for microspore maturation in Arabidopsis**. *Plant J* 2008, **55**(2):266-277.

11. Sorensen AM, Krober S, Unte US, Huijser P, Dekker K, Saedler H: **The Arabidopsis ABORTED MICROSPORES (AMS) gene encodes a MYC class transcription factor**. *Plant J* 2003, **33**(2):413-423.

12. Phan HA, Iacuone S, Li SF, Parish RW: **The MYB80 Transcription Factor Is Required for Pollen Development and the Regulation of Tapetal Programmed Cell Death in Arabidopsis thaliana**. *Plant Cell* 2011, **23**(6):2209-2224.

13. Yang C, Vizcay-Barrena G, Conner K, Wilson ZA: **MALE STERILITY1 is required for tapetal development and pollen wall biosynthesis**. *Plant Cell* 2007, **19**(11):3530-3548.

14. Hird DL, Worrall D, Hodge R, Smartt S, Paul W, Scott R: **The anther-specific protein encoded by the Brassica napus and Arabidopsis thaliana A6 gene displays similarity to beta-1,3-glucanases**. *Plant J* 1993, **4**(6):1023-1033.

15. Aarts MGM, Hodge R, Kalantidis K, Florack D, Wilson ZA, Mulligan BJ, Stiekema WJ, Scott R, Pereira A: **The Arabidopsis MALE STERILITY 2 protein shares similarity with reductases in elongation/condensation complexes**. *Plant J* 1997, **12**(3):615-623.

16. Dobritsa AA, Lei ZT, Nishikawa S, Urbanczyk-Wochniak E, Huhman DV, Preuss D, Sumner LW: **LAP5 and LAP6 Encode Anther-Specific Proteins with Similarity to Chalcone Synthase Essential for Pollen Exine Development in Arabidopsis**. *Plant Physiol* 2010, **153**(3):937-955.

17. de Azevedo Souza C, Kim SS, Koch S, Kienow L, Schneider K, McKim SM, Haughn GW, Kombrink E, Douglas CJ: **A novel fatty Acyl-CoA Synthetase is required for pollen development and sporopollenin biosynthesis in Arabidopsis**. *Plant Cell* 2009, **21**(2):507-525.

18. Matsuno M, Compagnon V, Schoch GA, Schmitt M, Debayle D, Bassard JE, Pollet B, Hehn A, Heintz D, Ullmann P *et al*: **Evolution of a novel phenolic pathway for pollen development**. *Science* 2009, **325**(5948):1688-1692.

19. Dobritsa AA, Shrestha J, Morant M, Pinot F, Matsuno M, Swanson R, Moller BL, Preuss D: **CYP704B1 is a long-chain fatty acid omega-hydroxylase essential for sporopollenin synthesis in pollen of Arabidopsis**. *Plant Physiol* 2009, **151**(2):574-589.

20. Quilichini TD, Friedmann MC, Samuels AL, Douglas CJ: **ATP-Binding Cassette Transporter G26 Is Required for Male Fertility and Pollen Exine Formation in Arabidopsis**. *Plant Physiol* 2010, **154**(2):678-690.

21. Rieu I, Ruiz-Rivero O, Fernandez-Garcia N, Griffiths J, Powers SJ, Gong F, Linhartova T, Eriksson S, Nilsson O, Thomas SG *et al*: **The gibberellin biosynthetic genes AtGA20ox1 and AtGA20ox2 act, partially redundantly, to promote growth and development throughout the Arabidopsis life cycle**. *Plant J* 2008, **53**(3):488-504.

22. Cheng YF, Dai XH, Zhao YD: **Auxin biosynthesis by the YUCCA flavin monooxygenases controls the formation of floral organs and vascular tissues in Arabidopsis**. *Genes & development* 2006, **20**(13):1790-1799.

23. Kaur J, Sebastian J, Siddiqi I: **The Arabidopsis-mei2-like genes play a role in meiosis and vegetative growth in Arabidopsis**. *Plant Cell* 2006, **18**(3):545-559.

24. Yang M, Hu Y, Lodhi M, McCombie WR, Ma H: **The Arabidopsis SKP1-LIKE1 gene is essential for male meiosis and may control homologue separation**. *Proc Natl Acad Sci U S A* 1999, **96**(20):11416-11421.

25. Bai XF, Peirson BN, Dong FG, Xue C, Makaroff CA: **Isolation and characterization of SYN1, a RAD21-like gene essential for meiosis in Arabidopsis**. *Plant Cell* 1999, **11**(3):417-430.

26. Chelysheva L, Diallo S, Vezon D, Gendrot G, Vrielynck N, Belcram K, Rocques N, Marquez-Lema A, Bhatt AM, Horlow C *et al*: **AtREC8 and AtSCC3 are essential to the monopolar orientation of the kinetochores during meiosis**. *J Cell Sci* 2005, **118**(20):4621-4632.

27. Armstrong SJ, Caryl AP, Jones GH, Franklin FC: **Asy1, a protein required for meiotic chromosome synapsis, localizes to axis-associated chromatin in Arabidopsis and Brassica**. *J Cell Sci* 2002, **115**(Pt 18):3645-3655.

28. Li WX, Chen CB, Markmann-Mulisch U, Timofejeva L, Schmelzer E, Ma H, Reiss B: **The Arabidopsis AtRAD51 gene is dispensable for vegetative development but required for meiosis**. *P Natl Acad Sci USA* 2004, **101**(29):10596-10601.

29. Bundock P, Hooykaas P: **Severe developmental defects, hypersensitivity to DNA-damaging agents, and lengthened telomeres in Arabidopsis MRE11 mutants**. *Plant Cell* 2002, **14**(10):2451-2462.

30. Kerzendorfer C, Vignard J, Pedrosa-Harand A, Siwiec T, Akimcheva S, Jolivet S, Sablowski R, Armstrong S, Schweizer D, Mercier R *et al*: **The Arabidopsis thaliana MND1 homologue plays a key role in meiotic homologous pairing, synapsis and recombination**. *J Cell Sci* 2006, **119**(12):2486-2496.

31. Keeney S, Giroux CN, Kleckner N: **Meiosis-specific DNA double-strand breaks are catalyzed by Spo11, a member of a widely conserved protein family**. *Cell* 1997, **88**(3):375-384.

32. Lu XD, Liu XL, An LZ, Zhang W, Sun J, Pei HJ, Meng HY, Fan YL, Zhang CY: **The Arabidopsis MutS homolog AtMSH5 is required for normal meiosis**. *Cell Res* 2008, **18**(5):589-599.

33. Hartung F, Suer S, Bergmann T, Puchta H: **The role of AtMUS81 in DNA repair and its genetic interaction with the helicase AtRecQ4A**. *Nucleic Acids Res* 2006, **34**(16):4438-4448.

34. Kim HJ, Oh SA, Brownfield L, Hong SH, Ryu H, Hwang I, Twell D, Nam HG: **Control of plant germline proliferation by SCF(FBL17) degradation of cell cycle inhibitors**. *Nature* 2008, **455**(7216):1134-1137.

35. Oh SA, Johnson A, Smertenko A, Rahman D, Park SK, Hussey PJ, Twell D: **A divergent cellular role for the FUSED kinase family in the plant-specific cytokinetic phragmoplast**. *Current Biology* 2005, **15**(23):2107-2111.

36. Park SK, Howden R, Twell D: **The Arabidopsis thaliana gametophytic mutation gemini pollen1 disrupts microspore polarity, division asymmetry and pollen cell fate**. *Development* 1998, **125**(19):3789-3799.

37. Guterman I, Shalit M, Menda N, Piestun D, Dafny-Yelin M, Shalev G, Bar E, Davydov O, Ovadis M, Emanuel M *et al*: **Rose scent: Genomics approach to discovering novel floral fragrance-related genes**. *Plant Cell* 2002, **14**(10):2325-2338.

38. Scalliet G, Journot N, Jullien F, Baudino S, Magnard JL, Channeliere S, Vergne P, Dumas C, Bendahmane M, Cock JM *et al*: **Biosynthesis of the major scent components 3,5-dimethoxytoluene and 1,3,5-trimethoxybenzene by novel rose O-methyltransferases**. *Febs Lett* 2002, **523**(1-3):PII S0014-5793(0002)02956-02953.

39. Wu S, Watanabe N, Mita S, Dohra H, Ueda Y, Shibuya M, Ebizuka Y: **The key role of phloroglucinol O-methyltransferase in the biosynthesis of Rosa chinensis volatile 1,3,5-trimethoxybenzene**. *Plant Physiol* 2004, **135**(1):95-102.

40. Huang FC, Horvath G, Molnar P, Turcsi E, Deli J, Schrader J, Sandmann G, Schmidt H, Schwab W: **Substrate promiscuity of RdCCD1, a carotenoid cleavage oxygenase from Rosa damascena**. *Phytochemistry* 2009, **70**(4):457-464.

41. Huang FC, Molnar P, Schwab W: **Cloning and functional characterization of carotenoid cleavage dioxygenase 4 genes**. *J Exp Bot* 2009, **60**(11):3011-3022.

42. Kaminaga Y, Schnepp J, Peel G, Kish CM, Ben-Nissan G, Weiss D, Orlova I, Lavie O, Rhodes D, Wood K *et al*: **Plant phenylacetaldehyde synthase is a bifunctional homotetrameric enzyme that catalyzes phenylalanine decarboxylation and oxidation**. *J Biol Chem* 2006, **281**(33):23357-23366.

43. Chen XM, Kobayashi H, Sakai M, Hirata H, Asai T, Ohnishi T, Baldermann S, Watanabe N: **Functional characterization of rose phenylacetaldehyde reductase (PAR), an enzyme involved in the biosynthesis of the scent compound 2-phenylethanol**. *J Plant Physiol* 2011, **168**(2):88-95.
