# Supplementary material for: Transcriptome database resource and gene expression atlas for the rose
Source: BMC Genomics. 2012 Nov 20;13:638. doi: 10.1186/1471-2164-13-638 (PMC3518227; doi:10.1186/1471-2164-13-638)
Supplement: Additional file 5 — Table S3. Primers used in qPCR validation. [file 1471-2164-13-638-S5.docx]

| Gene | *ROSAseq*  Accession | Forward primer | Reverse primer |
| --- | --- | --- | --- |
| *RcGAPDH* | RC030013 | GCTGGCAGGTATCCTTTCTG | GGCGACAATATCAGCCAAGT |
| *RcEF1* | BI978089 | GGTAAGGACCTTCACATC | CAGCCTCCTTCTCAAACCTCT |
| *RcTCTP* | BI978618 | TTGGTCTTTGCCTACTACAAAGAGG | AAGCCAGTTGCTACTTCTTAGCACT |
| *RcTub* | AF394915 | ATTGAGCGTCCCACCTACAC | AGCATGAAATGGATCCTTGG |
| *RcActin* | RC046406 | TCAAGGATTGGTGGACTTCAGT | ACCAGAGAACAAGAATGCAAGC |
| *RcAG* | RC018174 | ATCCATCAGTACTCGCGCCAT | TGCACCCTCCATTGAAGATGAA |
| *RcSHP* | RC018174 | ACAATCGCCAAGGGCAGA | CTGCACTATTATTTAGGAGAAGAGGC |
| *RcAP2* | RC001587 | ATGAGTGATCGACAACAATGGC | TGTGAAGGTGGTCTAATTTGCG |
| *RcAP3 (TM6-like)* | RC000216 | ACCCTAATAACCACGGCAACC | CAACCAATTAAGCAAGACGGAGAT |
| *RcEuB3* | RC000470 | TGACTTGAGCCTGGCATTTTG | CGTACATAGTACGTAAGAATGCAAGTGA |
| *RcPI* | RC000382 | TTACGTGTCCAGCCTAATCAGC | AGAAGGACGAGAGAACGCTCAC |
| *RcSEP3* | RC000799 | ATCCGATACAAGTTGTGACGGC | TCACCACACAGAGTTGCTTGC |
| *RcPAAS* | RC001532 | cgtgtgggttcatgtggat | ccgaaattctggacaaatgc |
| *RcOOMT* | RC001532 | GGAGGAAAAGAAAGGAATGAGAAAG | CAATGAGAGACCTTAAACCCGAA |
| *RcCHS* | RC009706 | ATGGGCACAAGACCACTGGA | AAGCAGCCACACTGTGAAGC |
| *RcCCD4* | RC002390 | CGAGGTTTTTGGTGATGGACG | CCCTCACAAACAGTCCGTGAA |
| *RcGDS* | RC000029 | TGGTTACTGTGATGATGATCTGCA | TCCTTGAACTTGTTGAACGTATCG |
| *RcDFR* | RC000643 | AGCTTGGAGGACATGTTTGTAGG | CTCTCATCAGCCTCCTGCTTT |
| *RcANS* | RC000516 | CTGTTCTACGGCGGCAAAT | TTGTACTTGCCGTTGCTCAGA |
| *RcSOC1* | RC004090 | TCCAAGTTCAGATGTTGAGACTGA | GATTATTGACACGAGCAGTTCTCC |
| *RcCLV1* | RC015603 | TCAGTGAATATCCCCTCGCA | GACCACTTCCCTCATTGTTGGT |
| *RcICK* | RC047800 | AATCCTCACTGCTTCTTCGCC | TGTAGACGGTTTCACAACTC |
| *RcPIP2* | RC003733 | ATGGATCTTCTGGGTCGGAC | CGGAATGAACCCAAAGCTTT |
| *RcCycB1* | RC015367 | ACACCCAAGCAGTTGTTCCTC | GTTCCCATTGTTCTTCTGTGCA |
| *RcCycB2* | RC024631 | AATGCCCAAAGGAATCTGAG | ATTCTTCATCCTCCTCCATCC |
| *RcSAG12* | RC000046 | GGCAATCCAATGAGTGTGAAGT | GGCCGAGTGTTTTGCTTAAGAA |

**Supplementary Table S3 :** Primers used in qPCR validation.
